# Stem cells: past, present, and future

**DOI:** 10.1186/s13287-019-1165-5

**Published:** 2019-02-26

**Authors:** Wojciech Zakrzewski, Maciej Dobrzyński, Maria Szymonowicz, Zbigniew Rybak

**Affiliations:** 1 Department of Experimental Surgery and Biomaterials Research, Wroclaw Medical University, Bujwida 44, Wrocław, 50-345 Poland; 2 Department of Conservative Dentistry and Pedodontics, Krakowska 26, Wrocław, 50-425 Poland

**Keywords:** Stem cells, Differentiation, Pluripotency, Induced pluripotent stem cell (iPSC), Teratoma formation assay, Stem cell derivation, Growth media, Tissue banks, Tissue transplantation

## Abstract

In recent years, stem cell therapy has become a very promising and advanced scientific research topic. The development of treatment methods has evoked great expectations. This paper is a review focused on the discovery of different stem cells and the potential therapies based on these cells. The genesis of stem cells is followed by laboratory steps of controlled stem cell culturing and derivation. Quality control and teratoma formation assays are important procedures in assessing the properties of the stem cells tested. Derivation methods and the utilization of culturing media are crucial to set proper environmental conditions for controlled differentiation. Among many types of stem tissue applications, the use of graphene scaffolds and the potential of extracellular vesicle-based therapies require attention due to their versatility. The review is summarized by challenges that stem cell therapy must overcome to be accepted worldwide. A wide variety of possibilities makes this cutting edge therapy a turning point in modern medicine, providing hope for untreatable diseases.

## Stem cell classification

Stem cells are unspecialized cells of the human body. They are able to differentiate into any cell of an organism and have the ability of self-renewal. Stem cells exist both in embryos and adult cells. There are several steps of specialization. Developmental potency is reduced with each step, which means that a unipotent stem cell is not able to differentiate into as many types of cells as a pluripotent one. This chapter will focus on stem cell classification to make it easier for the reader to comprehend the following chapters.

Totipotent stem cells are able to divide and differentiate into cells of the whole organism. Totipotency has the highest differentiation potential and allows cells to form both embryo and extra-embryonic structures. One example of a totipotent cell is a zygote, which is formed after a sperm fertilizes an egg. These cells can later develop either into any of the three germ layers or form a placenta. After approximately 4 days, the blastocyst’s inner cell mass becomes pluripotent. This structure is the source of pluripotent cells.

Pluripotent stem cells (PSCs) form cells of all germ layers but not extraembryonic structures, such as the placenta. Embryonic stem cells (ESCs) are an example. ESCs are derived from the inner cell mass of preimplantation embryos. Another example is induced pluripotent stem cells (iPSCs) derived from the epiblast layer of implanted embryos. Their pluripotency is a continuum, starting from completely pluripotent cells such as ESCs and iPSCs and ending on representatives with less potency—multi-, oligo- or unipotent cells. One of the methods to assess their activity and spectrum is the teratoma formation assay. iPSCs are artificially generated from somatic cells, and they function similarly to PSCs. Their culturing and utilization are very promising for present and future regenerative medicine.

Multipotent stem cells have a narrower spectrum of differentiation than PSCs, but they can specialize in discrete cells of specific cell lineages. One example is a haematopoietic stem cell, which can develop into several types of blood cells. After differentiation, a haematopoietic stem cell becomes an oligopotent cell. Its differentiation abilities are then restricted to cells of its lineage. However, some multipotent cells are capable of conversion into unrelated cell types, which suggests naming them pluripotent cells.

Oligopotent stem cells can differentiate into several cell types. A myeloid stem cell is an example that can divide into white blood cells but not red blood cells.

Unipotent stem cells are characterized by the narrowest differentiation capabilities and a special property of dividing repeatedly. Their latter feature makes them a promising candidate for therapeutic use in regenerative medicine. These cells are only able to form one cell type, e.g. dermatocytes.

## Stem cell biology

**Fig. 1:**
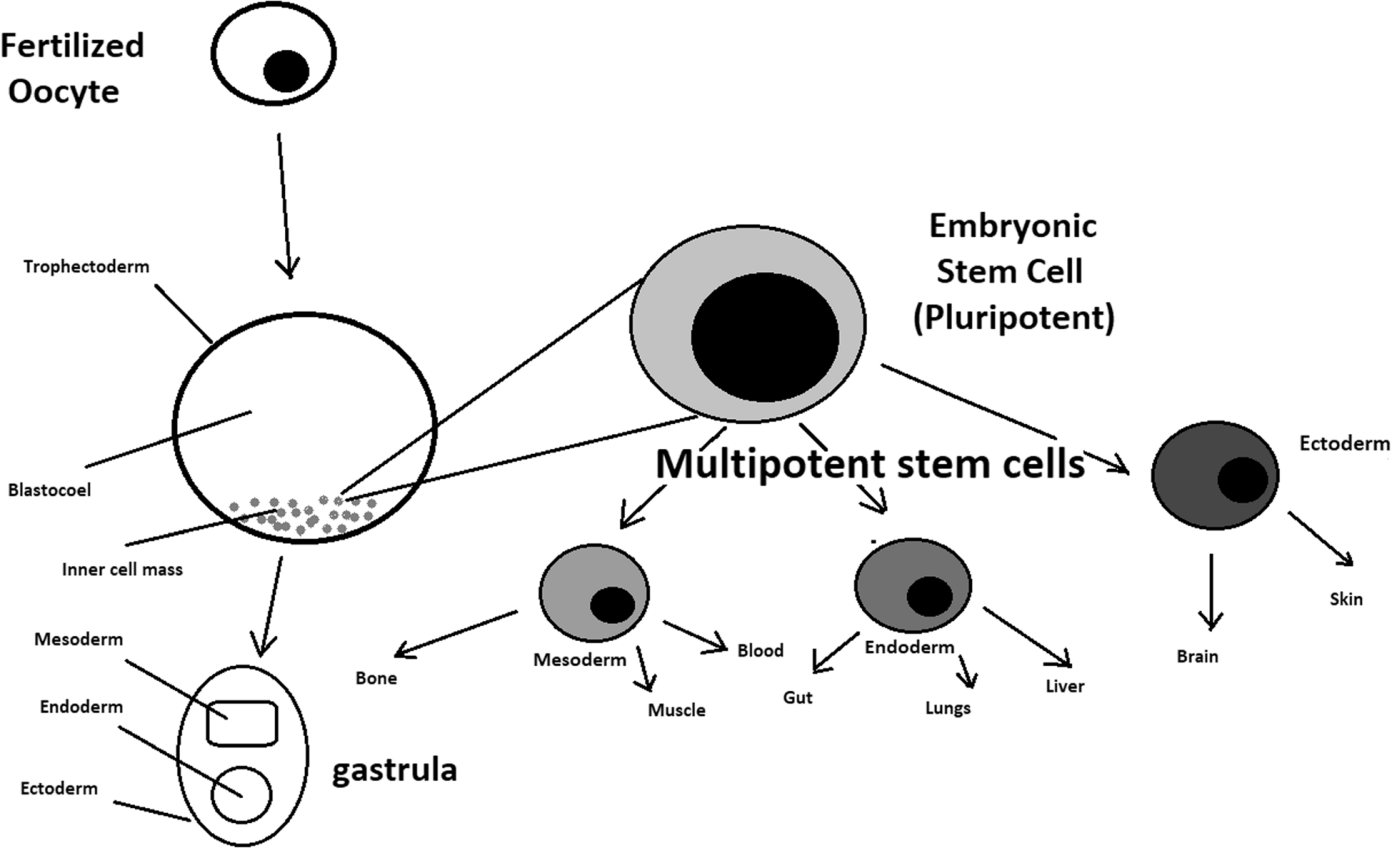
Oocyte development and formation of stem cells: the blastocoel, which is formed from oocytes, consists of embryonic stem cells that later differentiate into mesodermal, ectodermal, or endodermal cells. Blastocoel develops into the gastrula

A blastocyst is formed after the fusion of sperm and ovum fertilization. Its inner wall is lined with short-lived stem cells, namely, embryonic stem cells. Blastocysts are composed of two distinct cell types: the inner cell mass (ICM), which develops into epiblasts and induces the development of a foetus, and the trophectoderm (TE). Blastocysts are responsible for the regulation of the ICM microenvironment. The TE continues to develop and forms the extraembryonic support structures needed for the successful origin of the embryo, such as the placenta. As the TE begins to form a specialized support structure, the ICM cells remain undifferentiated, fully pluripotent and proliferative [1]. The pluripotency of stem cells allows them to form any cell of the organism. Human embryonic stem cells (hESCs) are derived from the ICM. During the process of embryogenesis, cells form aggregations called germ layers: endoderm, mesoderm and ectoderm (Fig. 1), each eventually giving rise to differentiated cells and tissues of the foetus and, later on, the adult organism [2]. After hESCs differentiate into one of the germ layers, they become multipotent stem cells, whose potency is limited to only the cells of the germ layer. This process is short in human development. After that, pluripotent stem cells occur all over the organism as undifferentiated cells, and their key abilities are proliferation by the formation of the next generation of stem cells and differentiation into specialized cells under certain physiological conditions.

Signals that influence the stem cell specialization process can be divided into external, such as physical contact between cells or chemical secretion by surrounding tissue, and internal, which are signals controlled by genes in DNA.

Stem cells also act as internal repair systems of the body. The replenishment and formation of new cells are unlimited as long as an organism is alive. Stem cell activity depends on the organ in which they are in; for example, in bone marrow, their division is constant, although in organs such as the pancreas, division only occurs under special physiological conditions.

### Stem cell functional division

#### Whole-body development

During division, the presence of different stem cells depends on organism development. Somatic stem cell ESCs can be distinguished. Although the derivation of ESCs without separation from the TE is possible, such a combination has growth limits. Because proliferating actions are limited, co-culture of these is usually avoided.

ESCs are derived from the inner cell mass of the blastocyst, which is a stage of pre-implantation embryo ca. 4 days after fertilization. After that, these cells are placed in a culture dish filled with culture medium. Passage is an inefficient but popular process of sub-culturing cells to other dishes. These cells can be described as pluripotent because they are able to eventually differentiate into every cell type in the organism. Since the beginning of their studies, there have been ethical restrictions connected to the medical use of ESCs in therapies. Most embryonic stem cells are developed from eggs that have been fertilized in an in vitro clinic, not from eggs fertilized in vivo.

Somatic or adult stem cells are undifferentiated and found among differentiated cells in the whole body after development. The function of these cells is to enable the healing, growth, and replacement of cells that are lost each day. These cells have a restricted range of differentiation options. Among many types, there are the following:

- Mesenchymal stem cells are present in many tissues. In bone marrow, these cells differentiate mainly into the bone, cartilage, and fat cells. As stem cells, they are an exception because they act pluripotently and can specialize in the cells of any germ layer.
- Neural cells give rise to nerve cells and their supporting cells—oligodendrocytes and astrocytes.
- Haematopoietic stem cells form all kinds of blood cells: red, white, and platelets.
- Skin stem cells form, for example, keratinocytes, which form a protective layer of skin.

The proliferation time of somatic stem cells is longer than that of ESCs. It is possible to reprogram adult stem cells back to their pluripotent state. This can be performed by transferring the adult nucleus into the cytoplasm of an oocyte or by fusion with the pluripotent cell. The same technique was used during cloning of the famous Dolly sheep.

**Fig. 2:**
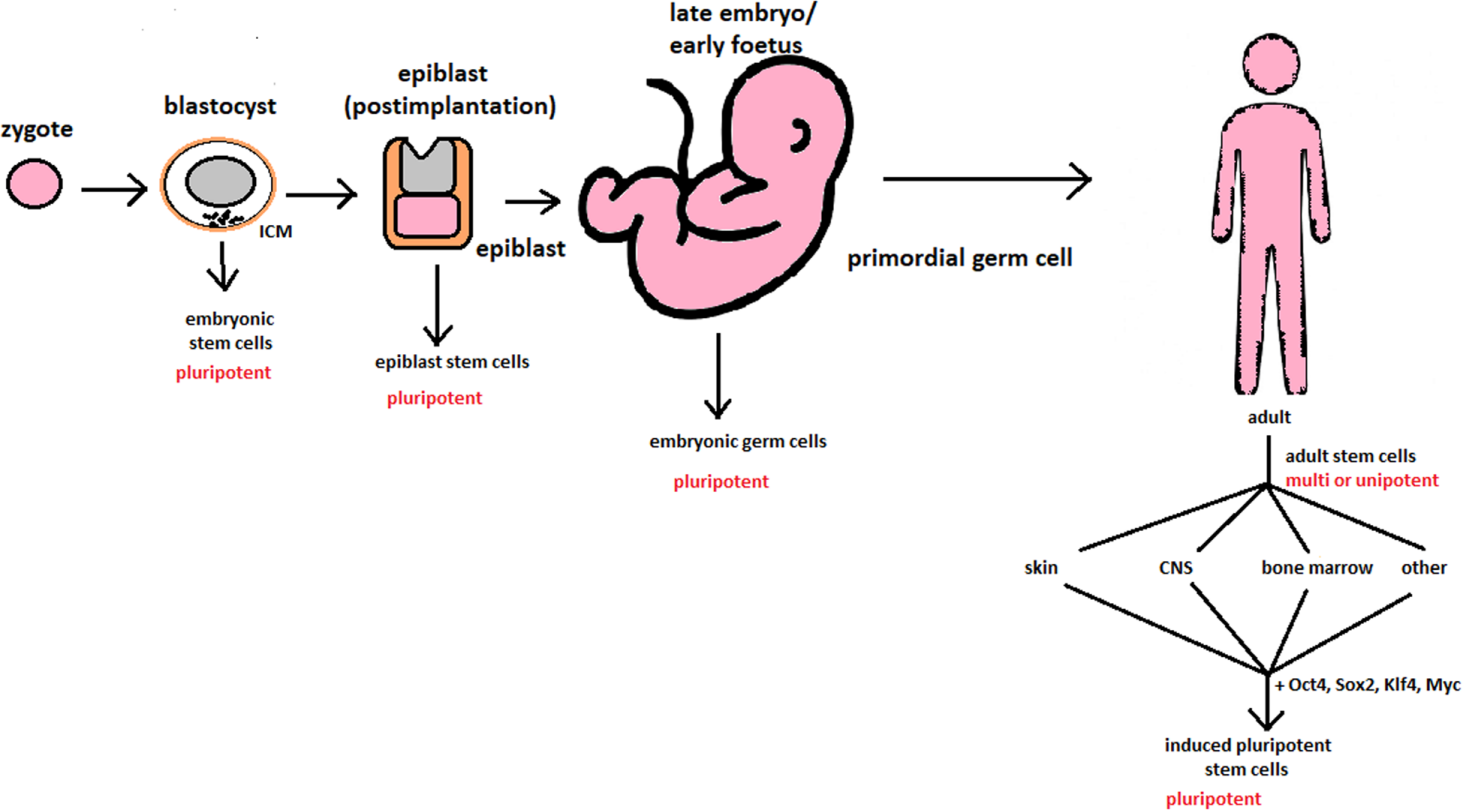
Changes in the potency of stem cells in human body development. Potency ranges from pluripotent cells of the blastocyst to unipotent cells of a specific tissue in a human body such as the skin, CNS, or bone marrow. Reversed pluripotency can be achieved by the formation of induced pluripotent stem cells using either octamer-binding transcription factor (Oct4), sex-determining region Y (Sox2), Kruppel-like factor 4 (Klf4), or the Myc gene

hESCs are involved in whole-body development. They can differentiate into pluripotent, totipotent, multipotent, and unipotent cells (Fig. 2) [2].

Pluripotent cells can be named totipotent if they can additionally form extraembryonic tissues of the embryo. Multipotent cells are restricted in differentiating to each cell type of given tissue. When tissue contains only one lineage of cells, stem cells that form them are called either called oligo- or unipotent.

## iPSC quality control and recognition by morphological differences

The comparability of stem cell lines from different individuals is needed for iPSC lines to be used in therapeutics [3]. Among critical quality procedures, the following can be distinguished:

Short tandem repeat analysis—This is the comparison of specific loci on the DNA of the samples. It is used in measuring an exact number of repeating units. One unit consists of 2 to 13 nucleotides repeating many times on the DNA strand. A polymerase chain reaction is used to check the lengths of short tandem repeats. The genotyping procedure of source tissue, cells, and iPSC seed and master cell banks is recommended.

Identity analysis—The unintentional switching of lines, resulting in other stem cell line contamination, requires rigorous assay for cell line identification.

Residual vector testing—An appearance of reprogramming vectors integrated into the host genome is hazardous, and testing their presence is a mandatory procedure. It is a commonly used procedure for generating high-quality iPSC lines. An acceptable threshold in high-quality research-grade iPSC line collections is ≤ 1 plasmid copies per 100 cells. During the procedure, 2 different regions, common to all plasmids, should be used as specific targets, such as EBNA and CAG sequences [3]. To accurately represent the test reactions, a standard curve needs to be prepared in a carrier of gDNA from a well-characterized hPSC line. For calculations of plasmid copies per cell, it is crucial to incorporate internal reference gDNA sequences to allow the quantification of, for example, ribonuclease P (RNaseP) or human telomerase reverse transcriptase (hTERT).

Karyotype—A long-term culture of hESCs can accumulate culture-driven mutations [4]. Because of that, it is crucial to pay additional attention to genomic integrity. Karyotype tests can be performed by resuscitating representative aliquots and culturing them for 48–72 h before harvesting cells for karyotypic analysis. If abnormalities are found within the first 20 karyotypes, the analysis must be repeated on a fresh sample. When this situation is repeated, the line is evaluated as abnormal. Repeated abnormalities must be recorded. Although karyology is a crucial procedure in stem cell quality control, the single nucleotide polymorphism (SNP) array, discussed later, has approximately 50 times higher resolution.

Viral testing—When assessing the quality of stem cells, all tests for harmful human adventitious agents must be performed (e.g. hepatitis C or human immunodeficiency virus). This procedure must be performed in the case of non-xeno-free culture agents.

Bacteriology—Bacterial or fungal sterility tests can be divided into culture- or broth-based tests. All the procedures must be recommended by pharmacopoeia for the jurisdiction in which the work is performed.

Single nucleotide polymorphism arrays—This procedure is a type of DNA microarray that detects population polymorphisms by enabling the detection of subchromosomal changes and the copy-neutral loss of heterozygosity, as well as an indication of cellular transformation. The SNP assay consists of three components. The first is labelling fragmented nucleic acid sequences with fluorescent dyes. The second is an array that contains immobilized allele-specific oligonucleotide (ASO) probes. The last component detects, records, and eventually interprets the signal.

Flow cytometry—This is a technique that utilizes light to count and profile cells in a heterogeneous fluid mixture. It allows researchers to accurately and rapidly collect data from heterogeneous fluid mixtures with live cells. Cells are passed through a narrow channel one by one. During light illumination, sensors detect light emitted or refracted from the cells. The last step is data analysis, compilation and integration into a comprehensive picture of the sample.

Phenotypic pluripotency assays—Recognizing undifferentiated cells is crucial in successful stem cell therapy. Among other characteristics, stem cells appear to have a distinct morphology with a high nucleus to cytoplasm ratio and a prominent nucleolus. Cells appear to be flat with defined borders, in contrast to differentiating colonies, which appear as loosely located cells with rough borders [5]. It is important that images of ideal and poor quality colonies for each cell line are kept in laboratories, so whenever there is doubt about the quality of culture, it can always be checked according to the representative image. Embryoid body formation or directed differentiation of monolayer cultures to produce cell types representative of all three embryonic germ layers must be performed. It is important to note that colonies cultured under different conditions may have different morphologies [6].

Histone modification and DNA methylation—Quality control can be achieved by using epigenetic analysis tools such as histone modification or DNA methylation. When stem cells differentiate, the methylation process silences pluripotency genes, which reduces differentiation potential, although other genes may undergo demethylation to become expressed [7]. It is important to emphasize that stem cell identity, together with its morphological characteristics, is also related to its epigenetic profile [8, 9]. According to Brindley [10], there is a relationship between epigenetic changes, pluripotency, and cell expansion conditions, which emphasizes that unmethylated regions appear to be serum-dependent.

## hESC derivation and media

**Fig. 3:**
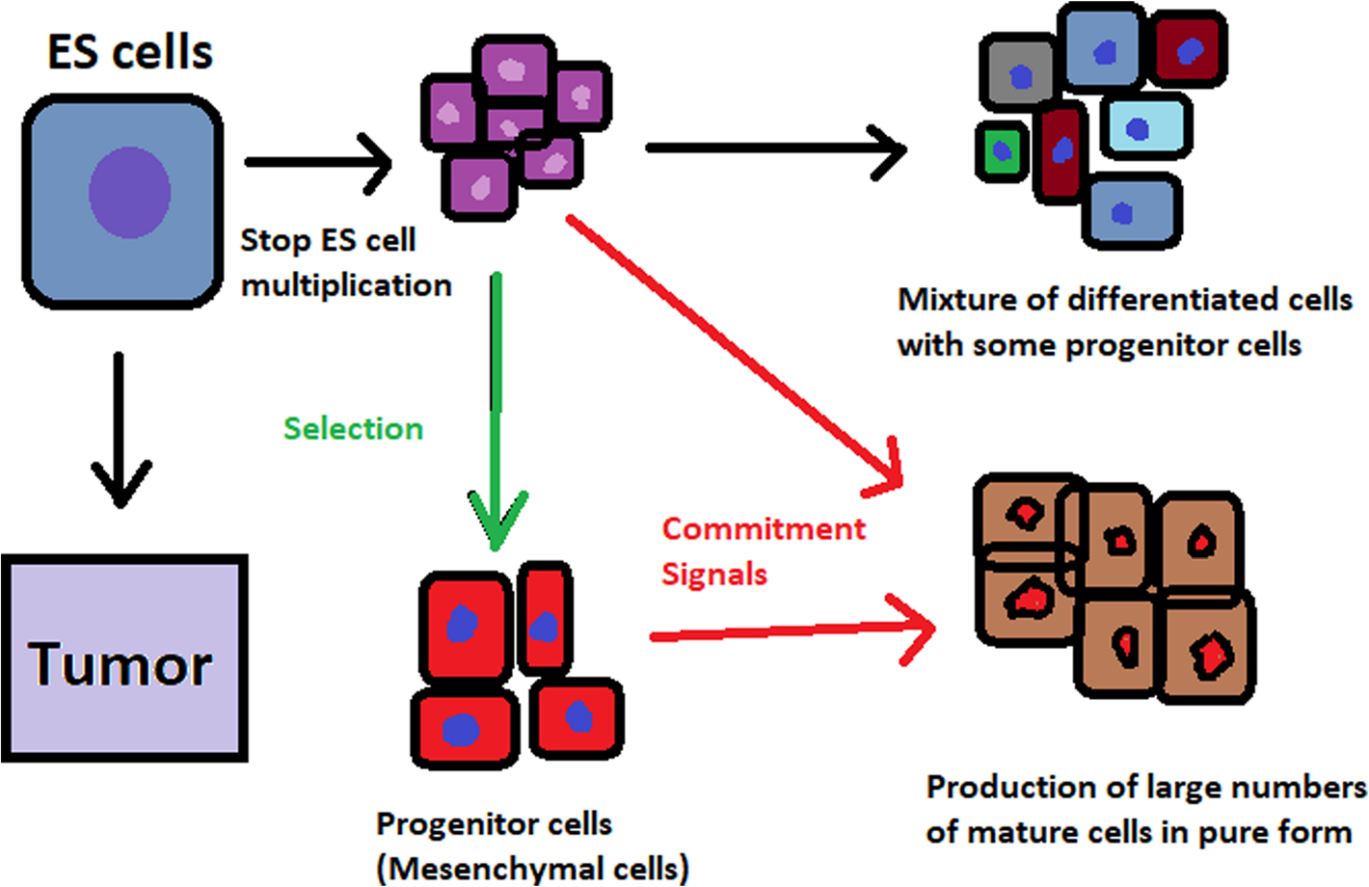
Spontaneous differentiation of hESCs causes the formation of a heterogeneous cell population. There is a different result, however, when commitment signals (in forms of soluble factors and culture conditions) are applied and enable the selection of progenitor cells

hESCs can be derived using a variety of methods, from classic culturing to laser-assisted methodologies or microsurgery [11]. hESC differentiation must be specified to avoid teratoma formation (see Fig. 3).

hESCs spontaneously differentiate into embryonic bodies (EBs) [12]. EBs can be studied instead of embryos or animals to predict their effects on early human development. There are many different methods for acquiring EBs, such as bioreactor culture [13], hanging drop culture [12], or microwell technology [14, 15]. These methods allow specific precursors to form in vitro [16].

**Fig. 4:**
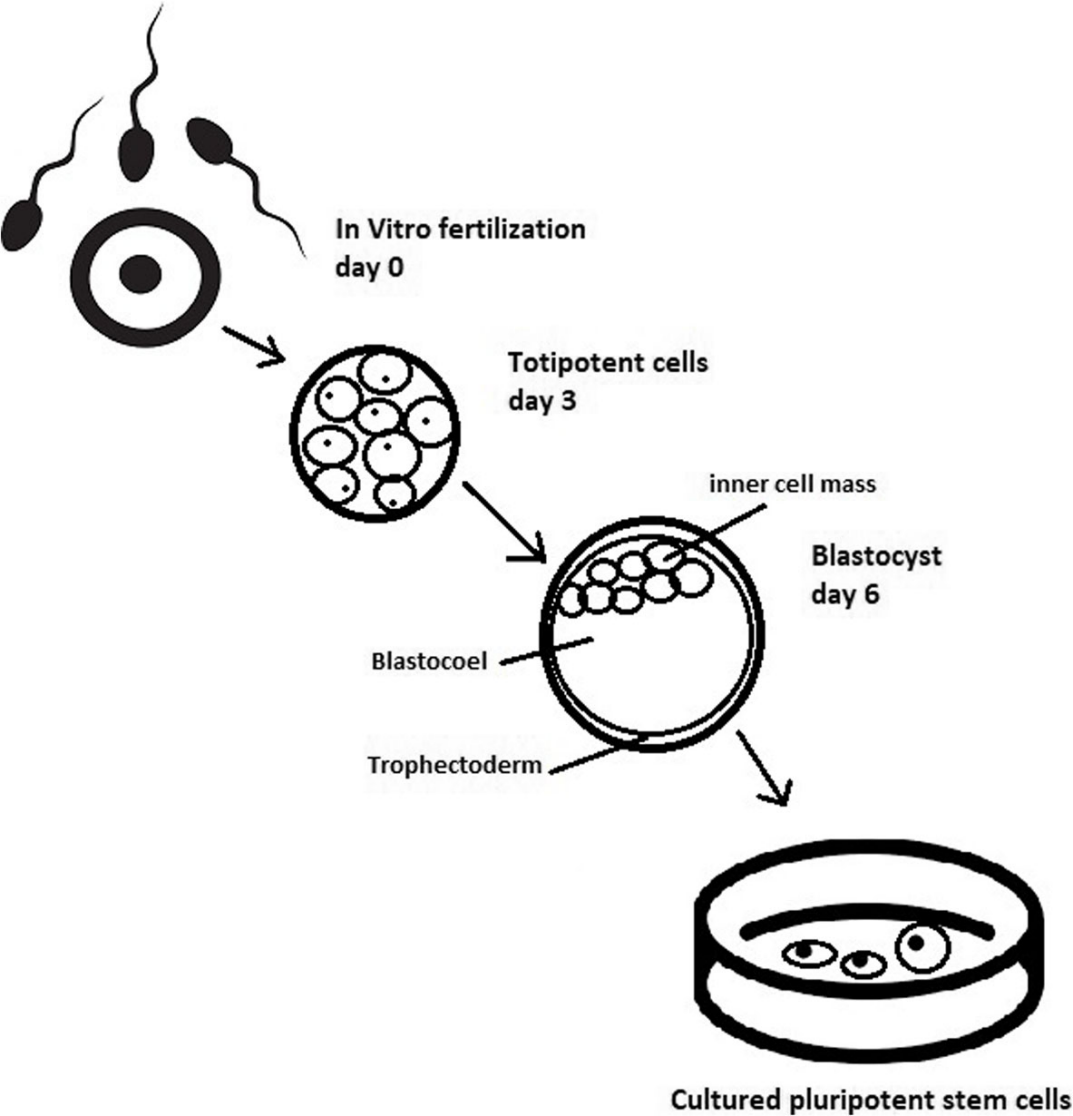
Culturing of pluripotent stem cells in vitro. Three days after fertilization, totipotent cells are formed. Blastocysts with ICM are formed on the sixth day after fertilization. Pluripotent stem cells from ICM can then be successfully transmitted on a dish

The essential part of these culturing procedures is a separation of inner cell mass to culture future hESCs (Fig. 4) [17]. Rosowski et al. [18] emphasizes that particular attention must be taken in controlling spontaneous differentiation. When the colony reaches the appropriate size, cells must be separated. The occurrence of pluripotent cells lasts for 1–2 days. Because the classical utilization of hESCs caused ethical concerns about gastrulas used during procedures, Chung et al. [19] found out that it is also possible to obtain hESCs from four cell embryos, leaving a higher probability of embryo survival. Additionally, Zhang et al. [20] used only in vitro fertilization growth-arrested cells.

Cell passaging is used to form smaller clusters of cells on a new culture surface [21]. There are four important passaging procedures.

*Enzymatic dissociation* is a cutting action of enzymes on proteins and adhesion domains that bind the colony. It is a gentler method than the manual passage. It is crucial to not leave hESCs alone after passaging. Solitary cells are more sensitive and can easily undergo cell death; collagenase type IV is an example [22, 23].

*Manual passage*, on the other hand, focuses on using cell scratchers. The selection of certain cells is not necessary. This should be done in the early stages of cell line derivation [24].

*Trypsin utilization* allows a healthy, automated hESC passage. Good Manufacturing Practice (GMP)-grade recombinant trypsin is widely available in this procedure [24]. However, there is a risk of decreasing the pluripotency and viability of stem cells [25]. Trypsin utilization can be halted with an inhibitor of the protein rho-associated protein kinase (ROCK) [26].

*Ethylenediaminetetraacetic acid* (*EDTA*) indirectly suppresses cell-to-cell connections by chelating divalent cations. Their suppression promotes cell dissociation [27].

Stem cells require a mixture of growth factors and nutrients to differentiate and develop. The medium should be changed each day.

Traditional culture methods used for hESCs are mouse embryonic fibroblasts (MEFs) as a feeder layer and bovine serum [28] as a medium. Martin et al. [29] demonstrated that hESCs cultured in the presence of animal products express the non-human sialic acid, *N*-glycolylneuraminic acid (NeuGc). Feeder layers prevent uncontrolled proliferation with factors such as leukaemia inhibitory factor (LIF) [30].

First feeder layer-free culture can be supplemented with serum replacement, combined with laminin [31]. This causes stable karyotypes of stem cells and pluripotency lasting for over a year.

Initial culturing media can be serum (e.g. foetal calf serum FCS), artificial replacement such as synthetic serum substitute (SSS), knockout serum replacement (KOSR), or StemPro [32]. The simplest culture medium contains only eight essential elements: DMEM/F12 medium, selenium, NaHCO_3,_ l-ascorbic acid, transferrin, insulin, TGFβ1, and FGF2 [33]. It is not yet fully known whether culture systems developed for hESCs can be allowed without adaptation in iPSC cultures.

## Turning point in stem cell therapy

**Fig. 5:**
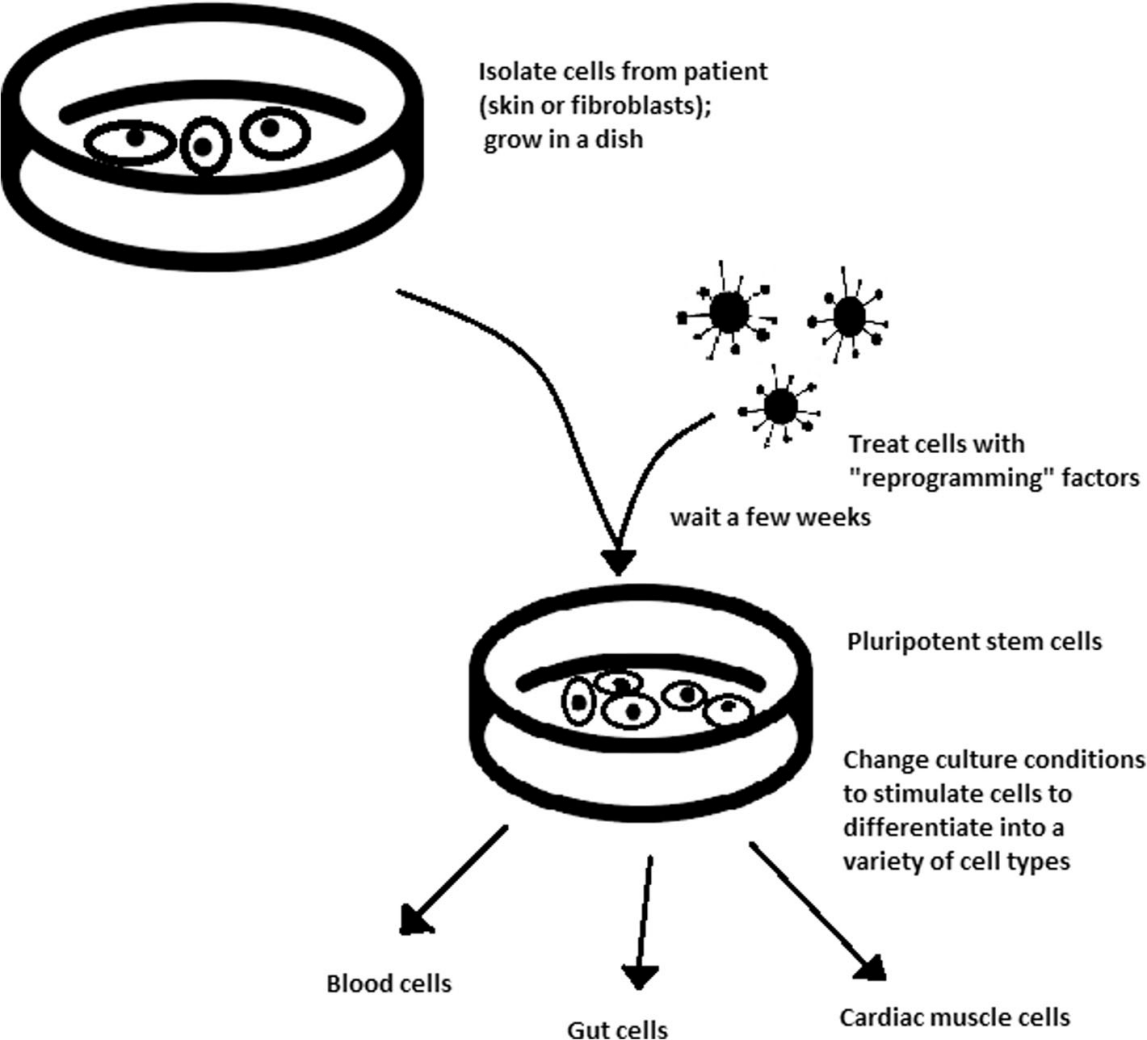
Retroviral-mediated transduction induces pluripotency in isolated patient somatic cells. Target cells lose their role as somatic cells and, once again, become pluripotent and can differentiate into any cell type of human body

The turning point in stem cell therapy appeared in 2006, when scientists Shinya Yamanaka, together with Kazutoshi Takahashi, discovered that it is possible to reprogram multipotent adult stem cells to the pluripotent state. This process avoided endangering the foetus’ life in the process. Retrovirus-mediated transduction of mouse fibroblasts with four transcription factors (Oct-3/4, Sox2, KLF4, and c-Myc) [34] that are mainly expressed in embryonic stem cells could induce the fibroblasts to become pluripotent (Fig. 5) [35]. This new form of stem cells was named iPSCs. One year later, the experiment also succeeded with human cells [36]. After this success, the method opened a new field in stem cell research with a generation of iPSC lines that can be customized and biocompatible with the patient. Recently, studies have focused on reducing carcinogenesis and improving the conduction system.

The turning point was influenced by former discoveries that happened in 1962 and 1987.

The former discovery was about scientist John Gurdon successfully cloning frogs by transferring a nucleus from a frog’s somatic cells into an oocyte. This caused a complete reversion of somatic cell development [37]. The results of his experiment became an immense discovery since it was previously believed that cell differentiation is a one-way street only, but his experiment suggested the opposite and demonstrated that it is even possible for a somatic cell to again acquire pluripotency [38].

The latter was a discovery made by Davis R.L. that focused on fibroblast DNA subtraction. Three genes were found that originally appeared in myoblasts. The enforced expression of only one of the genes, named myogenic differentiation 1 (Myod1), caused the conversion of fibroblasts into myoblasts, showing that reprogramming cells is possible, and it can even be used to transform cells from one lineage to another [39].

## iPSCs

Although pluripotency can occur naturally only in embryonic stem cells, it is possible to induce terminally differentiated cells to become pluripotent again. The process of direct reprogramming converts differentiated somatic cells into iPSC lines that can form all cell types of an organism. Reprogramming focuses on the expression of oncogenes such as Myc and Klf4 (Kruppel-like factor 4). This process is enhanced by a downregulation of genes promoting genome stability, such as p53. Additionally, cell reprogramming involves histone alteration. All these processes can cause potential mutagenic risk and later lead to an increased number of mutations. Quinlan et al. [40] checked fully pluripotent mouse iPSCs using whole genome DNA sequencing and structural variation (SV) detection algorithms. Based on those studies, it was confirmed that although there were single mutations in the non-genetic region, there were non-retrotransposon insertions. This led to the conclusion that current reprogramming methods can produce fully pluripotent iPSCs without severe genomic alterations.

During the course of development from pluripotent hESCs to differentiated somatic cells, crucial changes appear in the epigenetic structure of these cells. There is a restriction or permission of the transcription of genes relevant to each cell type. When somatic cells are being reprogrammed using transcription factors, all the epigenetic architecture has to be reconditioned to achieve iPSCs with pluripotency [41]. However, cells of each tissue undergo specific somatic genomic methylation. This influences transcription, which can further cause alterations in induced pluripotency [42].

### Source of iPSCs

Because pluripotent cells can propagate indefinitely and differentiate into any kind of cell, they can be an unlimited source, either for replacing lost or diseased tissues. iPSCs bypass the need for embryos in stem cell therapy. Because they are made from the patient’s own cells, they are autologous and no longer generate any risk of immune rejection.

At first, fibroblasts were used as a source of iPSCs. Because a biopsy was needed to achieve these types of cells, the technique underwent further research. Researchers investigated whether more accessible cells could be used in the method. Further, other cells were used in the process: peripheral blood cells, keratinocytes, and renal epithelial cells found in urine. An alternative strategy to stem cell transplantation can be stimulating a patient’s endogenous stem cells to divide or differentiate, occurring naturally when skin wounds are healing. In 2008, pancreatic exocrine cells were shown to be reprogrammed to functional, insulin-producing beta cells [43].

The best stem cell source appears to be the fibroblasts, which is more tempting in the case of logistics since its stimulation can be fast and better controlled [44].

#### Teratoma formation assay

The self-renewal and differentiation capabilities of iPSCs have gained significant interest and attention in regenerative medicine sciences. To study their abilities, a quality-control assay is needed, of which one of the most important is the teratoma formation assay. Teratomas are benign tumours. Teratomas are capable of rapid growth in vivo and are characteristic because of their ability to develop into tissues of all three germ layers simultaneously. Because of the high pluripotency of teratomas, this formation assay is considered an assessment of iPSC’s abilities [45].

Teratoma formation rate, for instance, was observed to be elevated in human iPSCs compared to that in hESCs [46]. This difference may be connected to different differentiation methods and cell origins. Most commonly, the teratoma assay involves an injection of examined iPSCs subcutaneously or under the testis or kidney capsule in mice, which are immune-deficient [47]. After injection, an immature but recognizable tissue can be observed, such as the kidney tubules, bone, cartilage, or neuroepithelium [30]. The injection site may have an impact on the efficiency of teratoma formation [48].

There are three groups of markers used in this assay to differentiate the cells of germ layers. For endodermal tissue, there is insulin/C-peptide and alpha-1 antitrypsin [49]. For the mesoderm, derivatives can be used, e.g. cartilage matrix protein for the bone and alcian blue for the cartilage. As ectodermal markers, class III B botulin or keratin can be used for keratinocytes.

Teratoma formation assays are considered the gold standard for demonstrating the pluripotency of human iPSCs, demonstrating their possibilities under physiological conditions. Due to their actual tissue formation, they could be used for the characterization of many cell lineages [50].

## Directed differentiation

To be useful in therapy, stem cells must be converted into desired cell types as necessary or else the whole regenerative medicine process will be pointless. Differentiation of ESCs is crucial because undifferentiated ESCs can cause teratoma formation in vivo. Understanding and using signalling pathways for differentiation is an important method in successful regenerative medicine. In directed differentiation, it is likely to mimic signals that are received by cells when they undergo successive stages of development [51]. The extracellular microenvironment plays a significant role in controlling cell behaviour. By manipulating the culture conditions, it is possible to restrict specific differentiation pathways and generate cultures that are enriched in certain precursors in vitro. However, achieving a similar effect in vivo is challenging. It is crucial to develop culture conditions that will allow the promotion of homogenous and enhanced differentiation of ESCs into functional and desired tissues.

Regarding the self-renewal of embryonic stem cells, Hwang et al. [52] noted that the ideal culture method for hESC-based cell and tissue therapy would be a defined culture free of either the feeder layer or animal components. This is because cell and tissue therapy requires the maintenance of large quantities of undifferentiated hESCs, which does not make feeder cells suitable for such tasks.

Most directed differentiation protocols are formed to mimic the development of an inner cell mass during gastrulation. During this process, pluripotent stem cells differentiate into ectodermal, mesodermal, or endodermal progenitors. Mall molecules or growth factors induce the conversion of stem cells into appropriate progenitor cells, which will later give rise to the desired cell type. There is a variety of signal intensities and molecular families that may affect the establishment of germ layers in vivo, such as fibroblast growth factors (FGFs) [53]; the Wnt family [54] or superfamily of transforming growth factors—β(TGFβ); and bone morphogenic proteins (BMP) [55]. Each candidate factor must be tested on various concentrations and additionally applied to various durations because the precise concentrations and times during which developing cells in embryos are influenced during differentiation are unknown. For instance, molecular antagonists of endogenous BMP and Wnt signalling can be used for ESC formation of ectoderm [56]. However, transient Wnt and lower concentrations of the TGFβ family trigger mesodermal differentiation [57]. Regarding endoderm formation, a higher activin A concentration may be required [58, 59].

There are numerous protocols about the methods of forming progenitors of cells of each of germ layers, such as cardiomyocytes [60], hepatocytes [61], renal cells [62], lung cells [63, 64], motor neurons [65], intestinal cells [66], or chondrocytes [67].

Directed differentiation of either iPSCs or ESCs into, e.g. hepatocytes, could influence and develop the study of the molecular mechanisms in human liver development. In addition, it could also provide the possibility to form exogenous hepatocytes for drug toxicity testing [68].

Levels of concentration and duration of action with a specific signalling molecule can cause a variety of factors. Unfortunately, for now, a high cost of recombinant factors is likely to limit their use on a larger scale in medicine. The more promising technique focuses on the use of small molecules. These can be used for either activating or deactivating specific signalling pathways. They enhance reprogramming efficiency by creating cells that are compatible with the desired type of tissue. It is a cheaper and non-immunogenic method.

One of the successful examples of small-molecule cell therapies is antagonists and agonists of the Hedgehog pathway. They show to be very useful in motor neuron regeneration [69]. Endogenous small molecules with their function in embryonic development can also be used in in vitro methods to induce the differentiation of cells; for example, retinoic acid, which is responsible for patterning the nervous system in vivo [70], surprisingly induced retinal cell formation when the laboratory procedure involved hESCs [71].

The efficacy of differentiation factors depends on functional maturity, efficiency, and, finally, introducing produced cells to their in vivo equivalent. Topography, shear stress, and substrate rigidity are factors influencing the phenotype of future cells [72].

The control of biophysical and biochemical signals, the biophysical environment, and a proper guide of hESC differentiation are important factors in appropriately cultured stem cells.

### Stem cell utilization and their manufacturing standards and culture systems

The European Medicines Agency and the Food and Drug Administration have set Good Manufacturing Practice (GMP) guidelines for safe and appropriate stem cell transplantation. In the past, protocols used for stem cell transplantation required animal-derived products [73].

The risk of introducing animal antigens or pathogens caused a restriction in their use. Due to such limitations, the technique required an obvious update [74]. Now, it is essential to use xeno-free equivalents when establishing cell lines that are derived from fresh embryos and cultured from human feeder cell lines [75]. In this method, it is crucial to replace any non-human materials with xeno-free equivalents [76].

NutriStem with LN-511, TeSR2 with human recombinant laminin (LN-511), and RegES with human foreskin fibroblasts (HFFs) are commonly used xeno-free culture systems [33]. There are many organizations and international initiatives, such as the National Stem Cell Bank, that provide stem cell lines for treatment or medical research [77].

### Stem cell use in medicine

Stem cells have great potential to become one of the most important aspects of medicine. In addition to the fact that they play a large role in developing restorative medicine, their study reveals much information about the complex events that happen during human development.

The difference between a stem cell and a differentiated cell is reflected in the cells’ DNA. In the former cell, DNA is arranged loosely with working genes. When signals enter the cell and the differentiation process begins, genes that are no longer needed are shut down, but genes required for the specialized function will remain active. This process can be reversed, and it is known that such pluripotency can be achieved by interaction in gene sequences. Takahashi and Yamanaka [78] and Loh et al. [79] discovered that octamer-binding transcription factor 3 and 4 (Oct3/4), sex determining region Y (SRY)-box 2 and Nanog genes function as core transcription factors in maintaining pluripotency. Among them, Oct3/4 and Sox2 are essential for the generation of iPSCs.

Many serious medical conditions, such as birth defects or cancer, are caused by improper differentiation or cell division. Currently, several stem cell therapies are possible, among which are treatments for spinal cord injury, heart failure [80], retinal and macular degeneration [81], tendon ruptures, and diabetes type 1 [82]. Stem cell research can further help in better understanding stem cell physiology. This may result in finding new ways of treating currently incurable diseases.

#### Haematopoietic stem cell transplantation

Haematopoietic stem cells are important because they are by far the most thoroughly characterized tissue-specific stem cell; after all, they have been experimentally studied for more than 50 years. These stem cells appear to provide an accurate paradigm model system to study tissue-specific stem cells, and they have potential in regenerative medicine.

Multipotent haematopoietic stem cell (HSC) transplantation is currently the most popular stem cell therapy. Target cells are usually derived from the bone marrow, peripheral blood, or umbilical cord blood [83]. The procedure can be autologous (when the patient’s own cells are used), allogenic (when the stem cell comes from a donor), or syngeneic (from an identical twin). HSCs are responsible for the generation of all functional haematopoietic lineages in blood, including erythrocytes, leukocytes, and platelets. HSC transplantation solves problems that are caused by inappropriate functioning of the haematopoietic system, which includes diseases such as leukaemia and anaemia. However, when conventional sources of HSC are taken into consideration, there are some important limitations. First, there is a limited number of transplantable cells, and an efficient way of gathering them has not yet been found. There is also a problem with finding a fitting antigen-matched donor for transplantation, and viral contamination or any immunoreactions also cause a reduction in efficiency in conventional HSC transplantations. Haematopoietic transplantation should be reserved for patients with life-threatening diseases because it has a multifactorial character and can be a dangerous procedure. iPSC use is crucial in this procedure. The use of a patient’s own unspecialized somatic cells as stem cells provides the greatest immunological compatibility and significantly increases the success of the procedure.

#### Stem cells as a target for pharmacological testing

Stem cells can be used in new drug tests. Each experiment on living tissue can be performed safely on specific differentiated cells from pluripotent cells. If any undesirable effect appears, drug formulas can be changed until they reach a sufficient level of effectiveness. The drug can enter the pharmacological market without harming any live testers. However, to test the drugs properly, the conditions must be equal when comparing the effects of two drugs. To achieve this goal, researchers need to gain full control of the differentiation process to generate pure populations of differentiated cells.

#### Stem cells as an alternative for arthroplasty

One of the biggest fears of professional sportsmen is getting an injury, which most often signifies the end of their professional career. This applies especially to tendon injuries, which, due to current treatment options focusing either on conservative or surgical treatment, often do not provide acceptable outcomes. Problems with the tendons start with their regeneration capabilities. Instead of functionally regenerating after an injury, tendons merely heal by forming scar tissues that lack the functionality of healthy tissues. Factors that may cause this failed healing response include hypervascularization, deposition of calcific materials, pain, or swelling [84].

Additionally, in addition to problems with tendons, there is a high probability of acquiring a pathological condition of joints called osteoarthritis (OA) [85]. OA is common due to the avascular nature of articular cartilage and its low regenerative capabilities [86]. Although arthroplasty is currently a common procedure in treating OA, it is not ideal for younger patients because they can outlive the implant and will require several surgical procedures in the future. These are situations where stem cell therapy can help by stopping the onset of OA [87]. However, these procedures are not well developed, and the long-term maintenance of hyaline cartilage requires further research.

Osteonecrosis of the femoral hip (ONFH) is a refractory disease associated with the collapse of the femoral head and risk of hip arthroplasty in younger populations [88]. Although total hip arthroplasty (THA) is clinically successful, it is not ideal for young patients, mostly due to the limited lifetime of the prosthesis. An increasing number of clinical studies have evaluated the therapeutic effect of stem cells on ONFH. Most of the authors demonstrated positive outcomes, with reduced pain, improved function, or avoidance of THA [89–91].

#### Rejuvenation by cell programming

Ageing is a reversible epigenetic process. The first cell rejuvenation study was published in 2011 [92]. Cells from aged individuals have different transcriptional signatures, high levels of oxidative stress, dysfunctional mitochondria, and shorter telomeres than in young cells [93]. There is a hypothesis that when human or mouse adult somatic cells are reprogrammed to iPSCs, their epigenetic age is virtually reset to zero [94]. This was based on an epigenetic model, which explains that at the time of fertilization, all marks of parenteral ageing are erased from the zygote’s genome and its ageing clock is reset to zero [95].

In their study, Ocampo et al. [96] used Oct4, Sox2, Klf4, and C-myc genes (OSKM genes) and affected pancreas and skeletal muscle cells, which have poor regenerative capacity. Their procedure revealed that these genes can also be used for effective regenerative treatment [97]. The main challenge of their method was the need to employ an approach that does not use transgenic animals and does not require an indefinitely long application. The first clinical approach would be preventive, focused on stopping or slowing the ageing rate. Later, progressive rejuvenation of old individuals can be attempted. In the future, this method may raise some ethical issues, such as overpopulation, leading to lower availability of food and energy.

For now, it is important to learn how to implement cell reprogramming technology in non-transgenic elder animals and humans to erase marks of ageing without removing the epigenetic marks of cell identity.

#### Cell-based therapies

**Fig. 6:**
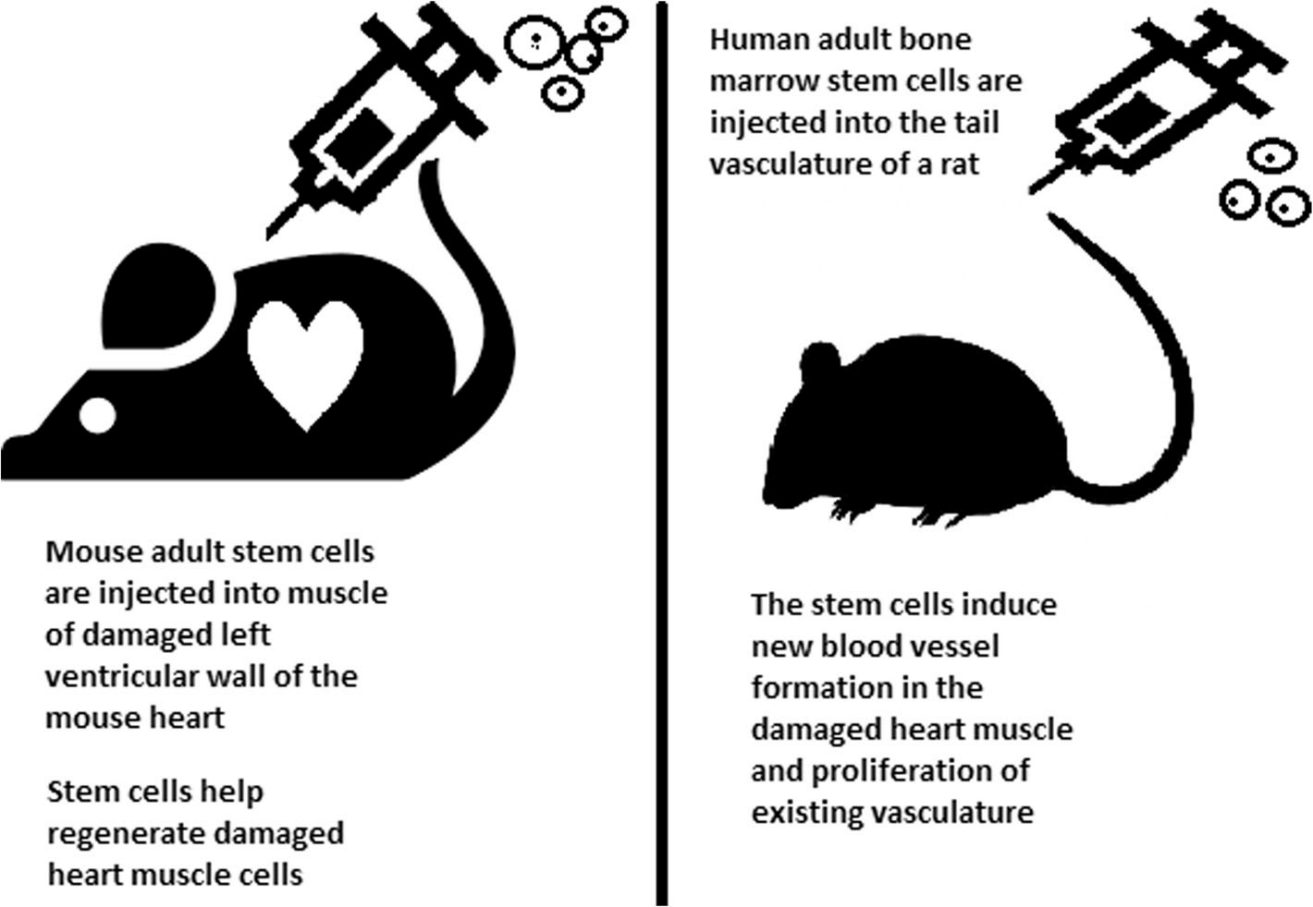
Stem cell experiments on animals. These experiments are one of the many procedures that proved stem cells to be a crucial factor in future regenerative medicine

Stem cells can be induced to become a specific cell type that is required to repair damaged or destroyed tissues (Fig. 6). Currently, when the need for transplantable tissues and organs outweighs the possible supply, stem cells appear to be a perfect solution for the problem. The most common conditions that benefit from such therapy are macular degenerations [98], strokes [99], osteoarthritis [89, 90], neurodegenerative diseases, and diabetes [100]. Due to this technique, it can become possible to generate healthy heart muscle cells and later transplant them to patients with heart disease.

In the case of type 1 diabetes, insulin-producing cells in the pancreas are destroyed due to an autoimmunological reaction. As an alternative to transplantation therapy, it can be possible to induce stem cells to differentiate into insulin-producing cells [101].

#### Stem cells and tissue banks

iPS cells with their theoretically unlimited propagation and differentiation abilities are attractive for the present and future sciences. They can be stored in a tissue bank to be an essential source of human tissue used for medical examination. The problem with conventional differentiated tissue cells held in the laboratory is that their propagation features diminish after time. This does not occur in iPSCs.

The umbilical cord is known to be rich in mesenchymal stem cells. Due to its cryopreservation immediately after birth, its stem cells can be successfully stored and used in therapies to prevent the future life-threatening diseases of a given patient.

Stem cells of human exfoliated deciduous teeth (SHED) found in exfoliated deciduous teeth has the ability to develop into more types of body tissues than other stem cells [102] (Table 1). Techniques of their collection, isolation, and storage are simple and non-invasive. Among the advantages of banking, SHED cells are:

- Guaranteed donor-match autologous transplant that causes no immune reaction and rejection of cells [103]
- Simple and painless for both child and parent
- Less than one third of the cost of cord blood storage
- Not subject to the same ethical concerns as embryonic stem cells [104]
- In contrast to cord blood stem cells, SHED cells are able to regenerate into solid tissues such as connective, neural, dental, or bone tissue [105, 106]
- SHED can be useful for close relatives of the donor

**Table 1:** Types of stem cells in human exfoliated deciduous teeth

SHED type	Role in regenerative medicine	References
Adipocytes	Heart muscle regeneration, cardiovascular disease prevention, treatment of spine and orthopaedic conditions, congestive heart failure, Crohn’s disease	[169–171]
Chondrocytes	Cartilage growth, suitable for transplants	[130, 172]
Osteoblasts	Bone tissues suitable for transplant, teeth growth, craniofacial defects, bone regeneration	[173, 174]
Mesenchymal	Spinal cord injury repair, restoration of feeling and movement in paralyzed patients, treatment of Alzheimer’s and Parkinson’s diseases	[103, 105, 121, 170, 175]

#### Fertility diseases

In 2011, two researchers, Katsuhiko Hayashi et al. [107], showed in an experiment on mice that it is possible to form sperm from iPSCs. They succeeded in delivering healthy and fertile pups in infertile mice. The experiment was also successful for female mice, where iPSCs formed fully functional eggs**.**

Young adults at risk of losing their spermatogonial stem cells (SSC), mostly cancer patients, are the main target group that can benefit from testicular tissue cryopreservation and autotransplantation. Effective freezing methods for adult and pre-pubertal testicular tissue are available [108].

Qiuwan et al. [109] provided important evidence that human amniotic epithelial cell (hAEC) transplantation could effectively improve ovarian function by inhibiting cell apoptosis and reducing inflammation in injured ovarian tissue of mice, and it could be a promising strategy for the management of premature ovarian failure or insufficiency in female cancer survivors.

For now, reaching successful infertility treatments in humans appears to be only a matter of time, but there are several challenges to overcome. First, the process needs to have high efficiency; second, the chances of forming tumours instead of eggs or sperm must be maximally reduced. The last barrier is how to mature human sperm and eggs in the lab without transplanting them to in vivo conditions, which could cause either a tumour risk or an invasive procedure.

#### Therapy for incurable neurodegenerative diseases

Thanks to stem cell therapy, it is possible not only to delay the progression of incurable neurodegenerative diseases such as Parkinson’s disease, Alzheimer’s disease (AD), and Huntington disease, but also, most importantly, to remove the source of the problem. In neuroscience, the discovery of neural stem cells (NSCs) has nullified the previous idea that adult CNS were not capable of neurogenesis [110, 111]. Neural stem cells are capable of improving cognitive function in preclinical rodent models of AD [112–114]. Awe et al. [115] clinically derived relevant human iPSCs from skin punch biopsies to develop a neural stem cell-based approach for treating AD. Neuronal degeneration in Parkinson’s disease (PD) is focal, and dopaminergic neurons can be efficiently generated from hESCs. PD is an ideal disease for iPSC-based cell therapy [116]. However, this therapy is still in an experimental phase (https://www.ncbi.nlm.nih.gov/pmc/articles/PMC4539501/). Brain tissue from aborted foetuses was used on patients with Parkinson’s disease [117]. Although the results were not uniform, they showed that therapies with pure stem cells are an important and achievable therapy.

#### Stem cell use in dentistry

Teeth represent a very challenging material for regenerative medicine. They are difficult to recreate because of their function in aspects such as articulation, mastication, or aesthetics due to their complicated structure. Currently, there is a chance for stem cells to become more widely used than synthetic materials. Teeth have a large advantage of being the most natural and non-invasive source of stem cells.

For now, without the use of stem cells, the most common periodontological treatments are either growth factors, grafts, or surgery. For example, there are stem cells in periodontal ligament [118, 119], which are capable of differentiating into osteoblasts or cementoblasts, and their functions were also assessed in neural cells [120]. Tissue engineering is a successful method for treating periodontal diseases. Stem cells of the root apical areas are able to recreate periodontal ligament. One of the possible methods of tissue engineering in periodontology is gene therapy performed using adenoviruses-containing growth factors [121].

As a result of animal studies, dentin regeneration is an effective process that results in the formation of dentin bridges [122].

Enamel is more difficult to regenerate than dentin. After the differentiation of ameloblastoma cells into the enamel, the former is destroyed, and reparation is impossible. Medical studies have succeeded in differentiating bone marrow stem cells into ameloblastoma [123].

**Fig. 7:**
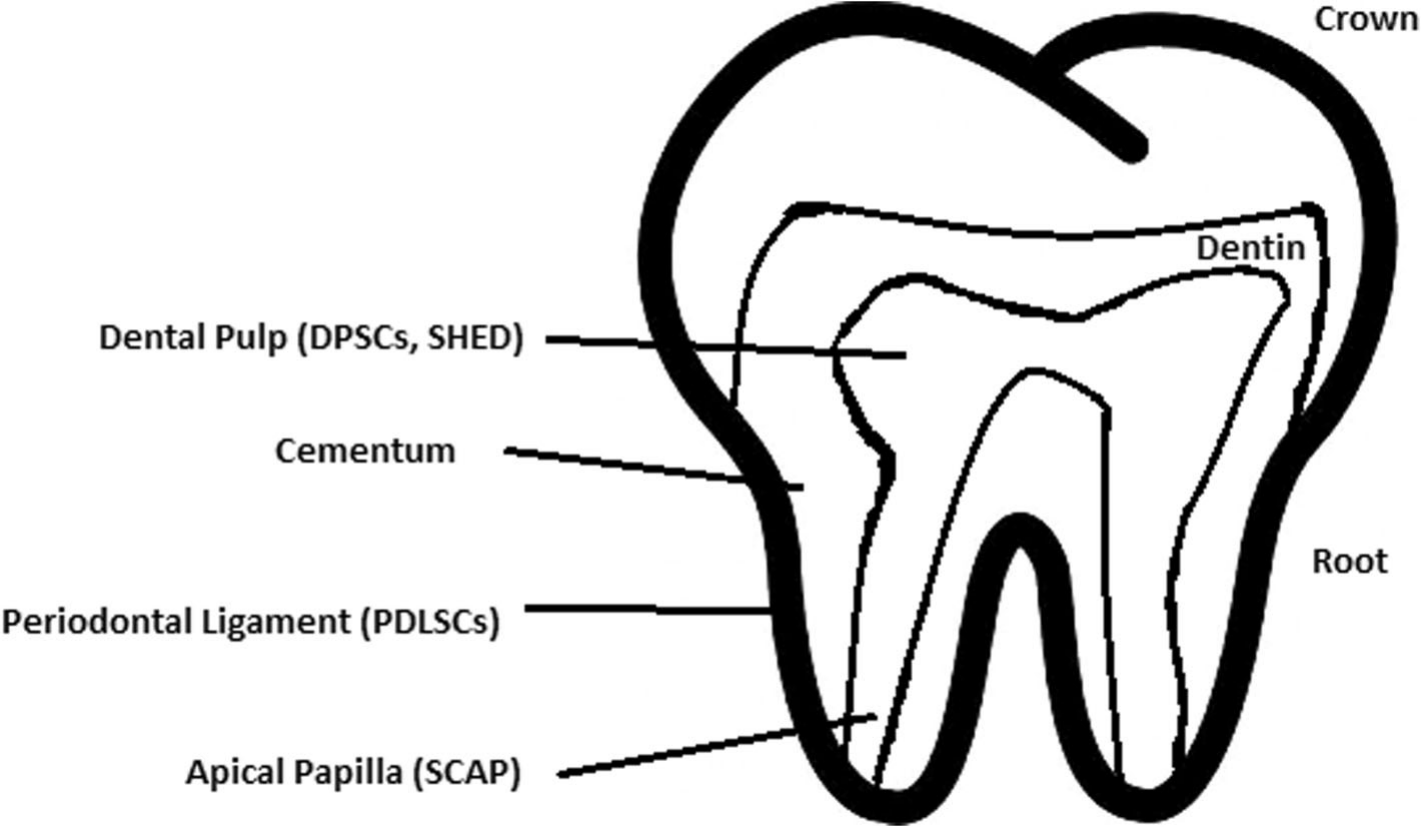
Localization of stem cells in dental tissues. Dental pulp stem cells (DPSCs) and human deciduous teeth stem cells (SHED) are located in the dental pulp. Periodontal ligaments stem cells are located in the periodontal ligament. Apical papilla consists of stem cells from the apical papilla (SCAP)

Healthy dental tissue has a high amount of regular stem cells, although this number is reduced when tissue is either traumatized or inflamed [124]. There are several dental stem cell groups that can be isolated (Fig. 7).

##### Dental pulp stem cell (DPSC)

These were the first dental stem cells isolated from the human dental pulp, which were [125] located inside dental pulp (Table 2). They have osteogenic and chondrogenic potential. Mesenchymal stem cells (MSCs) of the dental pulp, when isolated, appear highly clonogenic; they can be isolated from adult tissue (e.g. bone marrow, adipose tissue) and foetal (e.g. umbilical cord) [126] tissue, and they are able to differentiate densely [127]. MSCs differentiate into odontoblast-like cells and osteoblasts to form dentin and bone. Their best source locations are the third molars [125]. DPSCs are the most useful dental source of tissue engineering due to their easy surgical accessibility, cryopreservation possibility, increased production of dentin tissues compared to non-dental stem cells, and their anti-inflammatory abilities. These cells have the potential to be a source for maxillofacial and orthopaedic reconstructions or reconstructions even beyond the oral cavity. DPSCs are able to generate all structures of the developed tooth [128]. In particular, beneficial results in the use of DPSCs may be achieved when combined with other new therapies, such as periodontal tissue photobiomodulation (laser stimulation), which is an efficient technique in the stimulation of proliferation and differentiation into distinct cell types [129]. DPSCs can be induced to form neural cells to help treat neurological deficits.

**Table 2:** Detailed information about the differentiation of DPSCs and the studies connected to them [176]

	Cell type	Time	Differentiation strategy	Detection methods	References
Ectoderm	Odontogenic cells	4–8 weeks	Subcutaneous implantation	In vivo	[177–181]
	Schwann and neural cells	15 days	Factor-inducing	In vitro	[182]
Mesoderm	Osteocytes	3 weeks	Factor-inducing	In vitro	[183]
	Osteoblasts	3 months	Factor-inducing	In vitro	[184–186]
	Adipocytes	3 weeks	Factor-inducing	In vitro	[187–189]
	Myogenic cells	1 month	Factor-inducing	In vitro	[190]
	Chondrogenic cells	3 weeks	Factor-inducing co-cultured with human costal chondrocytes	In vitro	[191–196]
	Melanocytes	120 days	Factor-inducing	In vitro	[197]
Entoderm	Liver cells	40 days	Factor-inducing	In vitro	[198, 199]

Stem cells of human exfoliated deciduous teeth (SHED) have a faster rate of proliferation than DPSCs and differentiate into an even greater number of cells, e.g. other mesenchymal and non-mesenchymal stem cell derivatives, such as neural cells [130]. These cells possess one major disadvantage: they form a non-complete dentin/pulp-like complex in vivo. SHED do not undergo the same ethical concerns as embryonic stem cells. Both DPSCs and SHED are able to form bone-like tissues in vivo [131] and can be used for periodontal, dentin, or pulp regeneration. DPSCs and SHED can be used in treating, for example, neural deficits [132]. DPSCs alone were tested and successfully applied for alveolar bone and mandible reconstruction [133].

##### Periodontal ligament stem cells (PDLSCs)

These cells are used in periodontal ligament or cementum tissue regeneration. They can differentiate into mesenchymal cell lineages to produce collagen-forming cells, adipocytes, cementum tissue, Sharpey’s fibres, and osteoblast-like cells in vitro. PDLSCs exist both on the root and alveolar bone surfaces; however, on the latter, these cells have better differentiation abilities than on the former [134]. PDLSCs have become the first treatment for periodontal regeneration therapy because of their safety and efficiency [135, 136].

##### Stem cells from apical papilla (SCAP)

These cells are mesenchymal structures located within immature roots. They are isolated from human immature permanent apical papilla. SCAP are the source of odontoblasts and cause apexogenesis. These stem cells can be induced in vitro to form odontoblast-like cells, neuron-like cells, or adipocytes. SCAP have a higher capacity of proliferation than DPSCs, which makes them a better choice for tissue regeneration [137, 138].

##### Dental follicle stem cells (DFCs)

These cells are loose connective tissues surrounding the developing tooth germ. DFCs contain cells that can differentiate into cementoblasts, osteoblasts, and periodontal ligament cells [139, 140]. Additionally, these cells proliferate after even more than 30 passages [141]. DFCs are most commonly extracted from the sac of a third molar. When DFCs are combined with a treated dentin matrix, they can form a root-like tissue with a pulp-dentin complex and eventually form tooth roots [141]. When DFC sheets are induced by Hertwig’s epithelial root sheath cells, they can produce periodontal tissue; thus, DFCs represent a very promising material for tooth regeneration [142].

#### Pulp regeneration in endodontics

Dental pulp stem cells can differentiate into odontoblasts. There are few methods that enable the regeneration of the pulp.

The first is an ex vivo method. Proper stem cells are grown on a scaffold before they are implanted into the root channel [143].

The second is an in vivo method. This method focuses on injecting stem cells into disinfected root channels after the opening of the in vivo apex. Additionally, the use of a scaffold is necessary to prevent the movement of cells towards other tissues. For now, only pulp-like structures have been created successfully.

Methods of placing stem cells into the root channel constitute are either soft scaffolding [144] or the application of stem cells in apexogenesis or apexification. Immature teeth are the best source [145]. Nerve and blood vessel network regeneration are extremely vital to keep pulp tissue healthy.

The potential of dental stem cells is mainly regarding the regeneration of damaged dentin and pulp or the repair of any perforations; in the future, it appears to be even possible to generate the whole tooth. Such an immense success would lead to the gradual replacement of implant treatments. Mandibulary and maxillary defects can be one of the most complicated dental problems for stem cells to address.

#### Acquiring non-dental tissue cells by dental stem cell differentiation

In 2013, it was reported that it is possible to grow teeth from stem cells obtained extra-orally, e.g. from urine [146]. Pluripotent stem cells derived from human urine were induced and generated tooth-like structures. The physical properties of the structures were similar to natural ones except for hardness [127]. Nonetheless, it appears to be a very promising technique because it is non-invasive and relatively low-cost, and somatic cells can be used instead of embryonic cells. More importantly, stem cells derived from urine did not form any tumours, and the use of autologous cells reduces the chances of rejection [147].

#### Use of graphene in stem cell therapy

Over recent years, graphene and its derivatives have been increasingly used as scaffold materials to mediate stem cell growth and differentiation [148]. Both graphene and graphene oxide (GO) represent high in-plane stiffness [149]. Because graphene has carbon and aromatic network, it works either covalently or non-covalently with biomolecules; in addition to its superior mechanical properties, graphene offers versatile chemistry. Graphene exhibits biocompatibility with cells and their proper adhesion. It also tested positively for enhancing the proliferation or differentiation of stem cells [148]. After positive experiments, graphene revealed great potential as a scaffold and guide for specific lineages of stem cell differentiation [150]. Graphene has been successfully used in the transplantation of hMSCs and their guided differentiation to specific cells. The acceleration skills of graphene differentiation and division were also investigated. It was discovered that graphene can serve as a platform with increased adhesion for both growth factors and differentiation chemicals. It was also discovered that π-π binding was responsible for increased adhesion and played a crucial role in inducing hMSC differentiation [150].

#### Therapeutic potential of extracellular vesicle-based therapies

Extracellular vesicles (EVs) can be released by virtually every cell of an organism, including stem cells [151], and are involved in intercellular communication through the delivery of their mRNAs, lipids, and proteins. As Oh et al. [152] prove, stem cells, together with their paracrine factors—exosomes—can become potential therapeutics in the treatment of, e.g. skin ageing. Exosomes are small membrane vesicles secreted by most cells (30–120 nm in diameter) [153]. When endosomes fuse with the plasma membrane, they become exosomes that have messenger RNAs (mRNAs) and microRNAs (miRNAs), some classes of non-coding RNAs (IncRNAs) and several proteins that originate from the host cell [154]. IncRNAs can bind to specific loci and create epigenetic regulators, which leads to the formation of epigenetic modifications in recipient cells. Because of this feature, exosomes are believed to be implicated in cell-to-cell communication and the progression of diseases such as cancer [155]. Recently, many studies have also shown the therapeutic use of exosomes derived from stem cells, e.g. skin damage and renal or lung injuries [156].

In skin ageing, the most important factor is exposure to UV light, called “photoageing” [157], which causes extrinsic skin damage, characterized by dryness, roughness, irregular pigmentation, lesions, and skin cancers. In intrinsic skin ageing, on the other hand, the loss of elasticity is a characteristic feature. The skin dermis consists of fibroblasts, which are responsible for the synthesis of crucial skin elements, such as procollagen or elastic fibres. These elements form either basic framework extracellular matrix constituents of the skin dermis or play a major role in tissue elasticity. Fibroblast efficiency and abundance decrease with ageing [158]. Stem cells can promote the proliferation of dermal fibroblasts by secreting cytokines such as platelet-derived growth factor (PDGF), transforming growth factor β (TGF-β), and basic fibroblast growth factor. Huh et al. [159] mentioned that a medium of human amniotic fluid-derived stem cells (hAFSC) positively affected skin regeneration after longwave UV-induced (UVA, 315–400 nm) photoageing by increasing the proliferation and migration of dermal fibroblasts. It was discovered that, in addition to the induction of fibroblast physiology, hAFSC transplantation also improved diseases in cases of renal pathology, various cancers, or stroke [160, 161].

Oh [162] also presented another option for the treatment of skin wounds, either caused by physical damage or due to diabetic ulcers. Induced pluripotent stem cell-conditioned medium (iPSC-CM) without any animal-derived components induced dermal fibroblast proliferation and migration.

Natural cutaneous wound healing is divided into three steps: haemostasis/inflammation, proliferation, and remodelling. During the crucial step of proliferation, fibroblasts migrate and increase in number, indicating that it is a critical step in skin repair, and factors such as iPSC-CM that impact it can improve the whole cutaneous wound healing process. Paracrine actions performed by iPSCs are also important for this therapeutic effect [163]. These actions result in the secretion of cytokines such as TGF-β, interleukin (IL)-6, IL-8, monocyte chemotactic protein-1 (MCP-1), vascular endothelial growth factor (VEGF), platelet-derived growth factor-AA (PDGF-AA), and basic fibroblast growth factor (bFGF). Bae et al. [164] mentioned that TGF-β induced the migration of keratinocytes. It was also demonstrated that iPSC factors can enhance skin wound healing in vivo and in vitro when Zhou et al. [165] enhanced wound healing, even after carbon dioxide laser resurfacing in an in vivo study.

Peng et al. [166] investigated the effects of EVs derived from hESCs on in vitro cultured retinal glial, progenitor Müller cells, which are known to differentiate into retinal neurons. EVs appear heterogeneous in size and can be internalized by cultured Müller cells, and their proteins are involved in the induction and maintenance of stem cell pluripotency. These stem cell-derived vesicles were responsible for the neuronal trans-differentiation of cultured Müller cells exposed to them. However, the research article points out that the procedure was accomplished only on in vitro acquired retina.

### Challenges concerning stem cell therapy

Although stem cells appear to be an ideal solution for medicine, there are still many obstacles that need to be overcome in the future. One of the first problems is ethical concern.

**Fig. 8:**
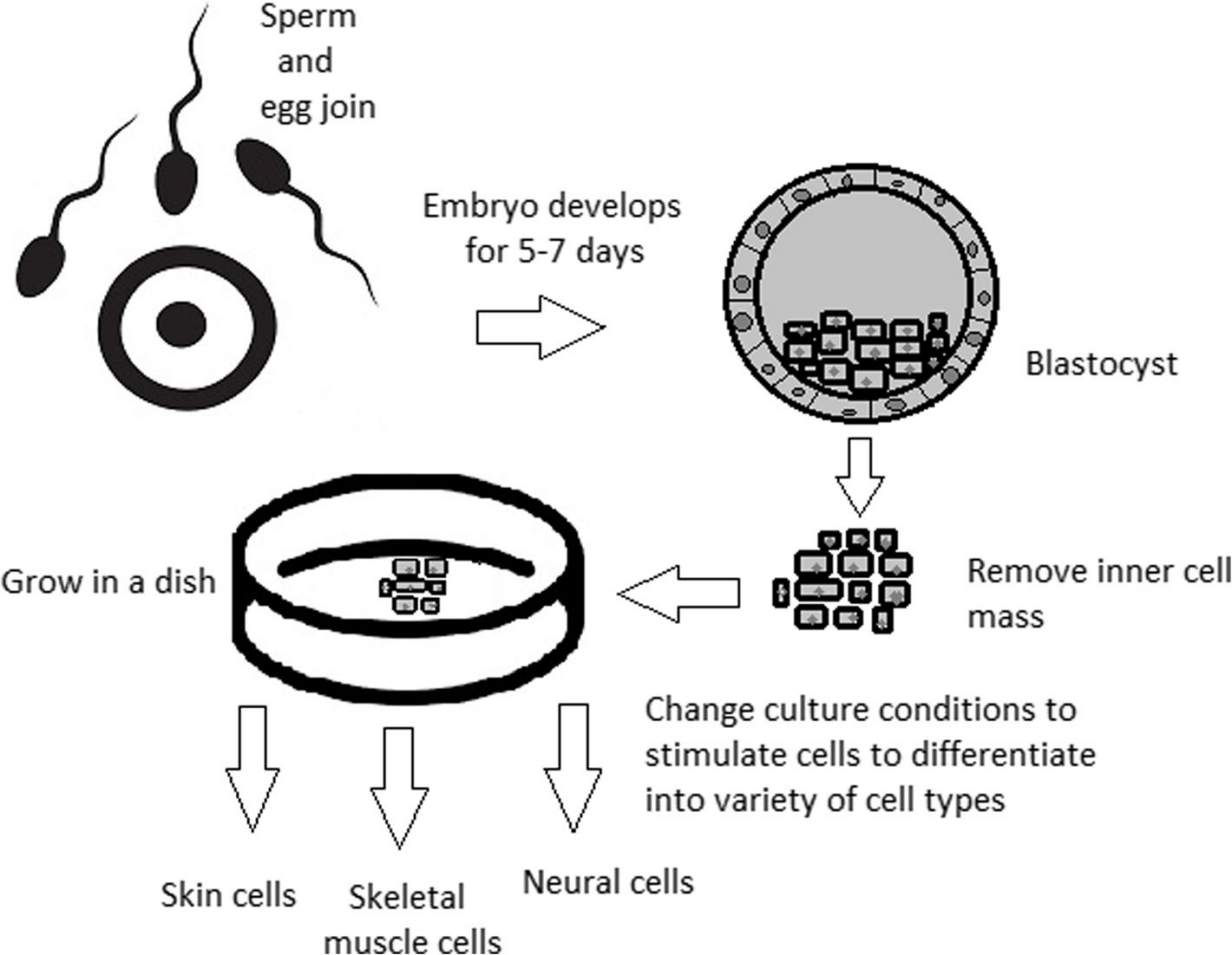
Use of inner cell mass pluripotent stem cells and their stimulation to differentiate into desired cell types

The most common pluripotent stem cells are ESCs. Therapies concerning their use at the beginning were, and still are, the source of ethical conflicts. The reason behind it started when, in 1998, scientists discovered the possibility of removing ESCs from human embryos. Stem cell therapy appeared to be very effective in treating many, even previously incurable, diseases. The problem was that when scientists isolated ESCs in the lab, the embryo, which had potential for becoming a human, was destroyed (Fig. 8). Because of this, scientists, seeing a large potential in this treatment method, focused their efforts on making it possible to isolate stem cells without endangering their source—the embryo.

For now, while hESCs still remain an ethically debatable source of cells, they are potentially powerful tools to be used for therapeutic applications of tissue regeneration. Because of the complexity of stem cell control systems, there is still much to be learned through observations in vitro. For stem cells to become a popular and widely accessible procedure, tumour risk must be assessed. The second problem is to achieve successful immunological tolerance between stem cells and the patient’s body. For now, one of the best ideas is to use the patient’s own cells and devolve them into their pluripotent stage of development.

New cells need to have the ability to fully replace lost or malfunctioning natural cells. Additionally, there is a concern about the possibility of obtaining stem cells without the risk of morbidity or pain for either the patient or the donor. Uncontrolled proliferation and differentiation of cells after implementation must also be assessed before its use in a wide variety of regenerative procedures on living patients [167].

One of the arguments that limit the use of iPSCs is their infamous role in tumourigenicity. There is a risk that the expression of oncogenes may increase when cells are being reprogrammed. In 2008, a technique was discovered that allowed scientists to remove oncogenes after a cell achieved pluripotency, although it is not efficient yet and takes a longer amount of time. The process of reprogramming may be enhanced by deletion of the tumour suppressor gene p53, but this gene also acts as a key regulator of cancer, which makes it impossible to remove in order to avoid more mutations in the reprogrammed cell. The low efficiency of the process is another problem, which is progressively becoming reduced with each year. At first, the rate of somatic cell reprogramming in Yamanaka’s study was up to 0.1%. The use of transcription factors creates a risk of genomic insertion and further mutation of the target cell genome. For now, the only ethically acceptable operation is an injection of hESCs into mouse embryos in the case of pluripotency evaluation [168].

## Stem cell obstacles in the future

Pioneering scientific and medical advances always have to be carefully policed in order to make sure they are both ethical and safe. Because stem cell therapy already has a large impact on many aspects of life, it should not be treated differently.

Currently, there are several challenges concerning stem cells. First, the most important one is about fully understanding the mechanism by which stem cells function first in animal models. This step cannot be avoided. For the widespread, global acceptance of the procedure, fear of the unknown is the greatest challenge to overcome.

The efficiency of stem cell-directed differentiation must be improved to make stem cells more reliable and trustworthy for a regular patient. The scale of the procedure is another challenge. Future stem cell therapies may be a significant obstacle. Transplanting new, fully functional organs made by stem cell therapy would require the creation of millions of working and biologically accurate cooperating cells. Bringing such complicated procedures into general, widespread regenerative medicine will require interdisciplinary and international collaboration.

The identification and proper isolation of stem cells from a patient’s tissues is another challenge. Immunological rejection is a major barrier to successful stem cell transplantation. With certain types of stem cells and procedures, the immune system may recognize transplanted cells as foreign bodies, triggering an immune reaction resulting in transplant or cell rejection.

One of the ideas that can make stem cells a “failsafe” is about implementing a self-destruct option if they become dangerous. Further development and versatility of stem cells may cause reduction of treatment costs for people suffering from currently incurable diseases. When facing certain organ failure, instead of undergoing extraordinarily expensive drug treatment, the patient would be able to utilize stem cell therapy. The effect of a successful operation would be immediate, and the patient would avoid chronic pharmacological treatment and its inevitable side effects.

Although these challenges facing stem cell science can be overwhelming, the field is making great advances each day. Stem cell therapy is already available for treating several diseases and conditions. Their impact on future medicine appears to be significant.

## Conclusion

After several decades of experiments, stem cell therapy is becoming a magnificent game changer for medicine. With each experiment, the capabilities of stem cells are growing, although there are still many obstacles to overcome. Regardless, the influence of stem cells in regenerative medicine and transplantology is immense. Currently, untreatable neurodegenerative diseases have the possibility of becoming treatable with stem cell therapy. Induced pluripotency enables the use of a patient’s own cells. Tissue banks are becoming increasingly popular, as they gather cells that are the source of regenerative medicine in a struggle against present and future diseases. With stem cell therapy and all its regenerative benefits, we are better able to prolong human life than at any time in history.

## Abbreviations

bFGF: Basic fibroblast growth factor
BMP: Bone morphogenic proteins
DFCs: Dental follicle stem cells
DPSC: Dental pulp stem cells
EBs: Embryonic bodies
ESCs: Embryonic stem cells
FGFs: Fibroblast growth factors
GMP: Good Manufacturing Practice
GO: Graphene oxide
hAFSC: Human amniotic fluid-derived stem cells
HESCs: Human embryonic stem cells
HFFs: Human foreskin fibroblasts
ICM: Inner cell mass
IncRNA: Non-coding RNA
iPSCs: Induced pluripotent stem cells
IVF: In vitro fertilization
KOSR: Knockout serum replacement
LIF: Leukaemia inhibitory factor
MCP-1: Monocyte chemotactic protein-1
MEFs: Fibroblasts
mRNA: Messenger RNA
MSCs: Mesenchymal stem cells of dental pulp
Myod1: Myogenic differentiation
OA: Osteoarthritis
Oct3/4: Octamer-binding transcription factor 3 and 4
PDGF: Platelet-derived growth factor
PDGF-AA: Platelet-derived growth factor-AA
PDLSCs: Periodontal ligament stem cells
ROCK: Rho-associated protein kinase
SCAP: Stem cells from apical papilla
SHED: Stem cells of human exfoliated deciduous teeth
SSS: Synthetic Serum Substitute
TE: Trophectoderm
VEGF: Vascular endothelial growth factor
β(TGFβ): Transforming growth factors
